# Inhibition of RhoA-Subfamily GTPases Suppresses Schwann Cell Proliferation Through Regulating AKT Pathway Rather Than ROCK Pathway

**DOI:** 10.3389/fncel.2018.00437

**Published:** 2018-11-20

**Authors:** Dandan Tan, Jinkun Wen, Lixia Li, Xianghai Wang, Changhui Qian, Mengjie Pan, Muhua Lai, Junyao Deng, Xiaofang Hu, Haowen Zhang, Jiasong Guo

**Affiliations:** ^1^Guangdong Provincial Key Laboratory of Construction and Detection in Tissue Engineering, Southern Medical University, Guangzhou, China; ^2^Department of Histology and Embryology, Southern Medical University, Guangzhou, China; ^3^Department of Histology and Embryology, Fujian University of Traditional Chinese Medicine, Fuzhou, China; ^4^Key Laboratory of Mental Health of the Ministry of Education, Southern Medical University, Guangzhou, China

**Keywords:** Schwann cell, proliferation, RhoA-subfamily GTPase, C3 transferase, AKT, ROCK

## Abstract

Inhibiting RhoA-subfamily GTPases by C3 transferase is widely recognized as a prospective strategy to enhance axonal regeneration. When C3 transferase is administered for treating the injured peripheral nerves, Schwann cells (SCs, important glial cells in peripheral nerve) are inevitably impacted and therefore SC bioeffects on nerve regeneration might be influenced. However, the potential role of C3 transferase on SCs remains elusive. Assessed by cell counting, EdU and water-soluble tetrazolium salt-1 (WST-1) assays as well as western blotting with PCNA antibody, herein we first found that CT04 (a cell permeable C3 transferase) treatment could significantly suppress SC proliferation. Unexpectedly, using Y27632 to inhibit ROCK (the well-accepted downstream signal molecule of RhoA subfamily) did not impact SC proliferation. Further studies indicated that CT04 could inactivate AKT pathway by altering the expression levels of phosphorylated AKT (p-AKT), PI3K and PTEN, while activating AKT pathway by IGF-1 or SC79 could reverse the inhibitory effect of CT04 on SC proliferation. Based on present data, we concluded that inhibition of RhoA-subfamily GTPases could suppress SC proliferation, and this effect is independent of conventional ROCK pathway but involves inactivation of AKT pathway.

## Introduction

Rho family small guanosine triphosphatases (Rho-GTPases) are essential in the regulation of diverse cellular functions such as regulation of actin cytoskeleton, vesicular trafficking and transcriptome dynamics (Hu and Selzer, 2017; Nomikou et al., 2017). Rho-GTPases cycle between an active GTP-bound and an inactive GDP-bound form, regulated by the opposing actions of guanine nucleotide exchange factors (GEFs) and GTPase-activating proteins (GAPs; Bai et al., 2015). The best characterized members of Rho family are classified into three subgroups, the RhoA (RhoA, B, C), Cdc42 (Cdc42, Tc10 and TcL) and Rac (Rac1, 2, 3 and RhoG) subfamilies, respectively (Erschbamer et al., 2005). RhoA subfamily is widely recognized as a crucial molecular switch to initiate growth cone collapse and inhibit axonal regrowth in the nervous system (Antoine-Bertrand et al., 2011; Matsukawa et al., 2018).

Peripheral nerve injury (PNI) is a common global clinical problem which involves approximate 2.8% clinic trauma patients, and it significantly affects the life quality of patients and arouses an enormous socioeconomic burden (Wang et al., 2017). Recently, inhibiting RhoA subfamily has been accepted as a prospective strategy to facilitate axonal regrowth and functional recovery after PNI (Hiraga et al., 2006; Auer et al., 2012; Hynds, 2015; Joshi et al., 2015). Due to C3 transferase is able to selectively inactivate RhoA-subfamily GTPases, it is widely used to promote neural regeneration (Auer et al., 2012, 2013; Zhou et al., 2012; Forgione and Fehlings, 2014; Gutekunst et al., 2016).

Peripheral nerves are composed not only of axons but also of Schwann cells (SCs), which wrap around the axons and form myelin sheath (Tricaud, 2017). SCs are the first cells activated following PNI and play vital roles in nerve regeneration through dedifferentiation and proliferation (Pan et al., 2017). The proliferated SCs can organize the clearance of broken axons and myelin debris by promoting macrophage recruitment or via phagocytosis by themselves, secrete neurotrophins to facilitate the axonal regrowth, form bands of Bunger in the distal stump to provide a permissive microenvironment for axon regeneration and ensuing remyelination (Monk et al., 2015; Jessen and Mirsky, 2016; Wong et al., 2017). Thus, SC proliferation is regarded as a crucial part of the nerve injury and regeneration (Jessen et al., 2015). When C3 transferase is administered to promote the axonal regeneration in the injured peripheral nerves, SCs are inevitably impacted and their bioeffects on nerve regeneration might be influenced. However, the potential roles of C3 transferase on SCs remain elusive. To figure out this issue, the present project was firstly designed to reveal the effect of CT04 (a cell permeable C3 transferase) on SC proliferation and then the underlying mechanisms were also studied.

## Materials and Methods

### Primary Cultures of Schwann Cells

The procedures of this study were performed in accordance with National Institutes of Health (NIH) guidelines for the care and use of laboratory animals (NIH Publications) and approved by the Animal Experimental Ethics Committee of the Southern Medical University (SMU), Guangdong Province, China. All efforts were made to minimize animal suffering and usage. Primary rat SCs were isolated and cultured according to our previous reports (Wen et al., 2017a,b). Briefly, spinal and sciatic nerves were aseptically isolated from 3-day to 5-day postnatal Sprague-Dawley (SD) rats (provided by the Experimental Animal Center of SMU). The collected nerves were dissociated by 0.25% Trypsin-EDTA (Gibco) at 37°C for 30 min and single cells were obtained by gentle pipetting. Following digestion and dissociation, the cells were centrifuged for 10 min at 1,000 rpm and re-suspended in DMEM/F12 (Corning) containing 10% fetal bovine serum (FBS, Corning). Then cells were plated onto poly-L-lysine (PLL, Sigma-Aldrich)-coated petri dish (Jet Biofil). The next day, 10 μM of cytosine arabinoside (Sigma-Aldrich) was added into the medium and incubated with the cells to eliminate fibroblasts. Forty-eight hours later, the medium was replaced by SC medium (DMEM/F12) containing 3% FBS, 3 μM forskolin (Sigma-Aldrich), 10 ng/ml heregulin (PeproTech) and 100 mg/ml penicillin-streptomycin (Gibco) to expand the cells. And all experiments of the present study were routinely performed using SCs collected at passages 3–5th. In designed experiments, 2 μg/ml CT04 (RhoA-subfamily GTPases inhibitor, Cytoskeleton), 50 μM Y27632 (ROCK inhibitor, Selleck), 150 ng/ml IGF-1 (AKT activator, PeproTech) or 20 μM SC79 (AKT activator, Selleck) was added into the culture medium and maintained for 24 h.

### Immunofluorescence Staining

To characterize the primary isolated cells, the cultured cells of passage 3 were fixed by 4% (***w/v***) paraformaldehyde for 20 min and washed three times with 0.01 M PBS. The fixed cells were permeabilized by 0.5% Triton X-100 (Sigma) for 30 min and then blocked with 5% bovine serum albumin (BSA, GBCBIO Technologies) in PBS for 1 h at room temperature, followed by the incubation with primary antibodies diluted in 1% BSA overnight at 4°C. The dilutions of the primary antibodies are as follows: rabbit anti-GFAP (1:400, Sigma-Aldrich); mouse anti-S100 (1:200, Millipore); and mouse anti-P75 (1:400, Millipore). Alexa 488 fluorescent conjugated secondary antibodies (1:400, Molecular Probes) were applied for 2 h at room temperature, and the nuclei were counterstained by 1 μg/ml 4′,6-diamidino-2-phenylindole (DAPI, Sigma) for 2 min. After immunofluorescence staining, the cultures were mounted using the anti-fading mounting medium (Vector) and images were captured with a fluorescent microscope (Leica).

### Schwann Cell Proliferation Assays

#### EdU Incorporation Assay

The EdU incorporation assay was conducted according to the manufacturer’s instructions (RiboBio). In brief, the cells were seeded at 1 × 10^4^/well in 96-well plates and incubated overnight to allow cell adherence. Cells were exposed to various drug treatments as designed for 24 h and then incubated with 50 μM EdU labeling reagent for 3 h prior to fixation. Following permeabilization in 0.5% Triton X-100, the cells underwent EdU staining. The cell nuclei were counterstained with DAPI. EdU-positive nuclei were determined under a fluorescence microscope (Leica). Five images were captured at the center and four quadrants in each plate using a fluorescent microscope. The EdU positive ratio was calculated as the number of EdU-positive cells divided by the number of total cells (positive for DAPI). Meanwhile, the cell density of each group was calculated and defined as the number of cells (positive for DAPI) in each captured image. The number of cells was counted using Image-Pro Plus software (Media Cybernetics).

#### WST-1 Assay

The cell proliferation was also evaluated by the water-soluble tetrazolium salt-1 (WST-1) assay using a Quick Cell Proliferation Assay Kit II (Abcam). SCs were prepared as described above (EdU incorporation assay). According to the manufacturer’s instructions, 10 μl of the WST reagent was added to each well of the 96-well plates for 3 h at 37°C. The absorbance was measured at 450 nm by using a microplate reader (BioTek). A_background_ is the absorbance of culture medium plus WST in the absence of cells. The actual absorbance of the control cells (A_control_) is the absorbance of A_control_ minus the absorbance of A_background_. The actual absorbance of the treated cells (A_experimental_) is the absorbance of A_experimental_ minus the absorbance of A_background_.

### Western Blotting

For western blotting, SCs were treated with various drugs as designed for 24 h. The subjected cells were washed twice with ice-cold PBS, scraped and lysed in RIPA buffer (GBCBIO) containing protease inhibitor cocktail (1:100, Cell Signaling). Lysates were incubated on ice for 30 min and centrifuged (14,000 rpm, 20 min, 4°C) in order to collect the supernatant. Extracts were combined with SDS-PAGE sample buffer (400 mM Tris/HCl, pH 6.8, 10% SDS, 50% glycerol, 500 mM DTT, 2 μg/ml bromophenol blue) and denatured by boiling for 10 min. Equal protein samples were loaded and resolved by 10% SDS-PAGE gels and transferred to polyvinylidene difluoride (PVDF) membrane (Bio-Rad) by liquid transfer. Membranes were blocked with 5% BSA for 1 h at room temperature and incubated with primary antibodies overnight at 4°C. The following primary antibodies were used in western blotting: rabbit anti-GAPDH (1:3,000, Multi Science); rabbit anti-PCNA (1:500, Cell Signaling Technology); rabbit anti-PTEN (1:1,000, Cell Signaling Technology); rabbit anti-PI3K (1:1,000, Cell Signaling Technology); rabbit anti-phospho-AKT (1:1,000, Cell Signaling Technology); rabbit anti-AKT (1:1,000, Cell Signaling Technology). After washed three times with Tris-buffered saline containing 0.05% Tween-20, the membranes were incubated with respective HRP-conjugated secondary antibodies (1:2,000, Cell Signaling Technology) for 2 h at room temperature. Immunoreactive proteins bands were detected and imaged by enhanced chemiluminescence (ECL, Millipore) using Lumazone system (Roper). After exposure, membranes were washed by stripping buffer (Thermo) for further incubation of another antibody if needed. Integrated optical density (IOD) of each lane was quantified with Image-Pro Plus software and the expression levels of targeted proteins were normalized by the IOD of GAPDH.

### Cytotoxicity Assay by Live/Dead Cell Staining

In order to assess whether the effect of CT04 on cell proliferation is attributed to the cytotoxicity of CT04, an assay with Live/Dead cell staining kit (BestBio) was conducted according to the manufacturer’s instructions. In brief, the cells were seeded at 1 × 10^4^/well in 96-well plates and incubated overnight to allow cell adherence. The culture media were supplemented with 2 μg/ml CT04 or 30% DMSO for 24 h and then incubated for 30 min with the Live/Dead cell staining solution which was prepared with the reagents A, B and C of the kit. After that, the cultures were observed with a fluorescence microscope to detect the live cells (stained by Calcein-AM with green fluorescence) and dead cells (stained by PI with red fluorescence). Five images were captured at the center and four quadrants in each culture. Then the live cell ratio was calculated as the number of Calcein-AM positive cells divided by the number of total cells (Calcein-AM-positive cells plus PI-positive cells).

### Statistical Analysis

All statistical analyses and calculations were carried out using SPSS 20.0 software (IBM, Armonk, NY, USA). Statistics for multiple comparisons were generated using one-way ANOVA followed by Bonferroni *post hoc* tests. Independent samples *t-test* was used to analyze values between two groups. All statistical graphs were plotted with the Graph Pad Prism 6.0 (Graph Pad software), and quantitative data were presented as mean ± standard deviation (SD). Differences were considered statistically significant with *P*-value < 0.05.

## Results

### CT04 Suppresses Schwann Cell Proliferation

**Figure 1:**
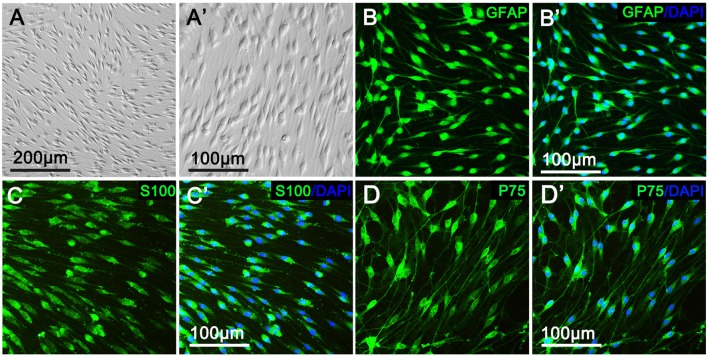
Characterization and identification of primary cultured Schwann cells (SCs).** (A,A′)** Primary cultured SCs showed a bipolar spindle shape under phase contrast microscope. More than 95% of the cells were positive for the SC specific markers, including GFAP **(B,B′)**, S100 **(C,C′)** and P75 **(D,D′)**.

**Figure 2:**
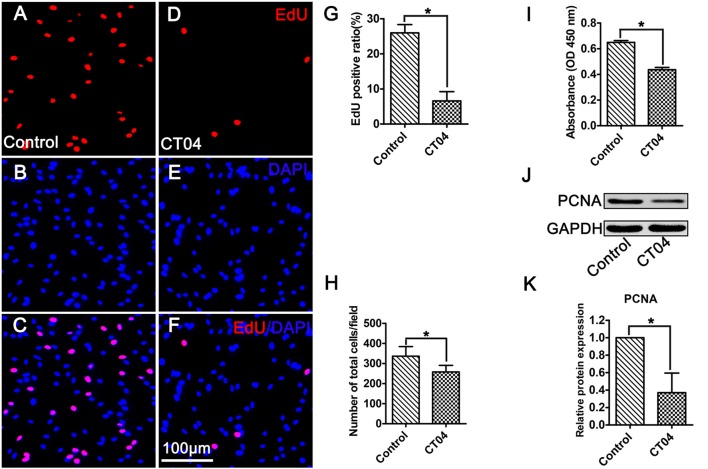
CT04 suppresses SC proliferation. **(A–G)** EdU staining showed that EdU positive ratio was suppressed by CT04 treatment (*n* = 15, *P < 0.05). **(H)** Statistical graph of cell density indicated that the number of total cells (positive for 4′,6-diamidino-2-phenylindole (DAPI)) was decreased in the presence of CT04 (*n* = 15, *P < 0.05). **(I)** Water-soluble tetrazolium salt-1 (WST-1) measurement revealed that CT04 markedly decreased the absorbance value compared to the control group (*n* = 4, *P < 0.05). **(J,K)** Western blotting displayed that the expression of PCNA was remarkably down-regulated in the CT04 treated cells (*n* = 6, *P < 0.05). The blots were cropped from different parts of the same gel. The expression level of PCNA in the control group was normalized to 1.

As shown in Figure 1, primary cultured SCs exhibited a bipolar spindle shape (Figures 1A,A′) and were positive for SC-specific markers, including GFAP, S100 and P75 (Figures 1B–D,B′–D′). To illustrate the effect of CT04 on SC proliferation, SCs were treated by CT04 for 24 h. Then, the EdU and WST-1 measurements were conducted. The EdU assay displayed that the ratio of EdU positive cells was significantly reduced in the presence of CT04 (Figures 2A–G). Furthermore, the statistical analysis of cell density indicated that the number of total cells was also decreased by CT04 treatment (Figure 2H). The WST-1 results revealed that CT04 markedly decreased the absorbance value compared to the control group (Figure 2I). Meanwhile, the level of PCNA, determined using western blotting, was remarkably down-regulated by CT04 treatment (Figures 2J,K). Taken together, all these results confirm that CT04 can suppress SC proliferation.

**Figure 3:**
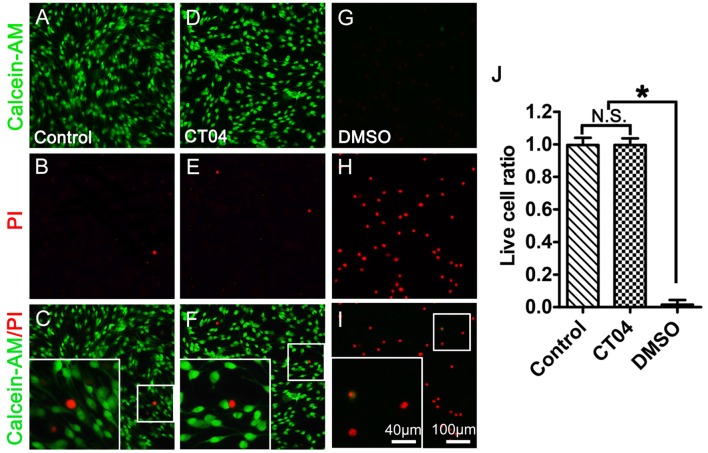
CT04 does not induce toxic effect on SC. The cytotoxicity of CT04 was evaluated using Live/Dead cell staining. **(A–J)** The Live/Dead cell staining and statistical diagrams suggested that addition of CT04 did not induce cell death in the SCs cultures (*n* = 20, *P < 0.05). N.S. as non-significance.

Further experiment with Live/Dead cell staining assay was designed to test the potential cytotoxicity of CT04 in the SC cultures. In order to confirm the efficiency and reliability of this assay, we set up a positive control (30% DMSO treatment) and a negative control (no drug treatment) to do the parallel experiment with the CT04 treatment. The results illustrated that the overwhelming majority of cells in both of the negative control group and CT04 group were live cells, only very few of cells in these two groups were dead cells (Figures 3A–F,J). However, almost all cells in the positive control group were dead cells (Figures 3G–J). These results demonstrate that the effect of CT04 on SC proliferation is not caused by cytotoxicity.

### Inhibition of ROCK Does Not Affect Schwann Cell Proliferation

**Figure 4:**
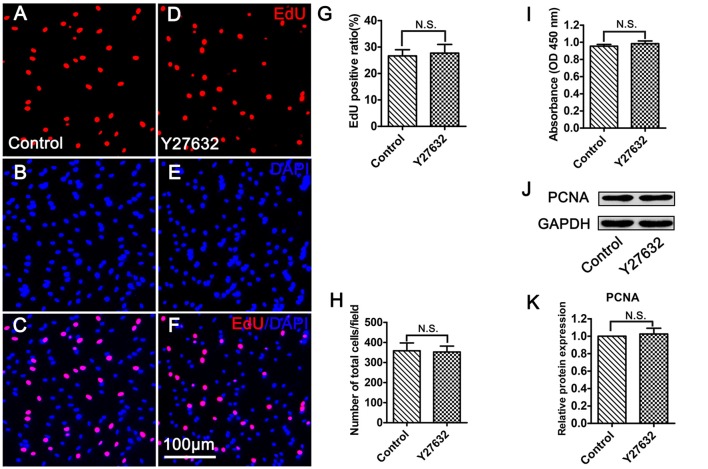
Inhibition of ROCK does not affect SC proliferation. **(A–G)** EdU assay showed that EdU positive ratio was not affected by Y27632 treatment (*n* = 15). **(H)** Statistical diagram of cell density suggested that the number of total cells was not altered in the presence of Y27632 (*n* = 15). **(I)** WST-1 measurement revealed that there was no significant difference in the absorbance value between the control group and Y27632 group (*n* = 4). **(J,K**) Western blotting indicated that the expression of PCNA was unaffected in the Y27632 treated cells (*n* = 6). The blots were cropped from different parts of the same gel. The expression level of PCNA in the control group was normalized to 1. N.S. as non-significance.

To identify whether CT04 modulates SC proliferation through ROCK which is the most well-known downstream effector of RhoA-subfamily GTPases, the SCs were treated with Y27632 (a widely used specific ROCK inhibitor) for 24 h. Unexpectedly, Y27632 did not result in the same effect on SC proliferation as CT04. As shown in Figure 4, the EdU assay indicated that the ratio of EdU positive cells as well as the cell density was not affected in the presence of Y27632 (Figures 4A–G,H). Meanwhile, WST-1 assay showed no difference in absorbance value between Y27632 and control groups (Figure 4I). In addition, the expression level of PCNA was similar between two groups (Figures 4J,K). These data strongly implicate that ROCK is not involved in the regulation of SC proliferation.

### CT04 Inactivates AKT Signaling Pathway

**Figure 5:**
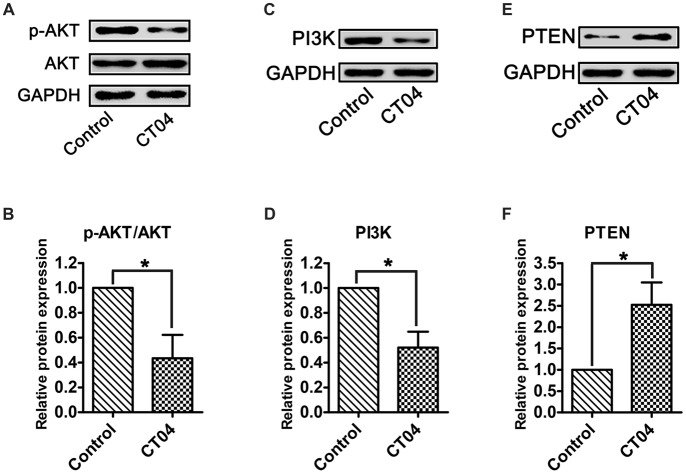
CT04 regulates the AKT signaling pathway. **(A,B)** The western blots indicated that the phosphorylation of AKT was markedly decreased in the presence of CT04 (*n* = 12, *P < 0.05). **(C–F)** Immunoblot assays and statistical diagrams also revealed that addition of CT04 resulted in down-regulation of PI3K and up-regulation of PTEN (*n* = 4, *P < 0.05). The blots were cropped from different parts of the same gel. The expression levels of target proteins in the control group were normalized to 1.

According to previous reports (He et al., 2011; Chen et al., 2016; Wu et al., 2016), AKT pathway is one of the most important pathways involved in regulating SC proliferation. To determine whether this pathway is responsible for mediating the CT04-induced suppression on SC proliferation, the total AKT, phosphorylated AKT (p-AKT) as well as AKT’s critical upstream regulator—PI3K (Gaesser and Fyffe-Maricich, 2016) and its crucial inhibitor—PTEN (Liu et al., 2017) were detected. Western blotting verified that the p-AKT was significantly decreased in CT04 group, while the total AKT was unaffected (Figures 5A,B). Furthermore, the expression of PI3K was down-regulated in the presence of CT04 (Figures 5C,D), while the expression of PTEN was up-regulated (Figures 5E,F). These data drew us to hypothesize that AKT pathway might be involved in the inhibitory effect of CT04 on SC proliferation.

### Activation of AKT Reverses the Inhibitory Effect of CT04 on SC Proliferation

**Figure 6:**
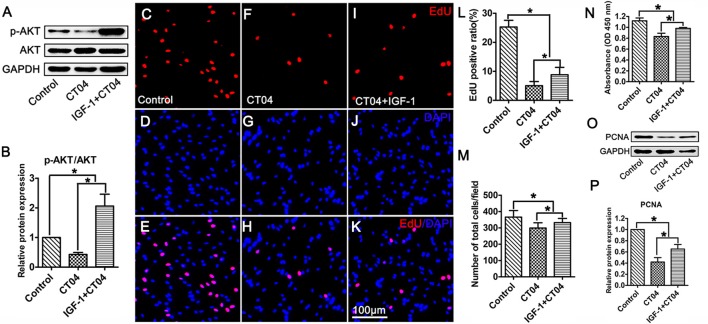
Activation of AKT by IGF-1 reverses the inhibitory effect of CT04 on SC proliferation. The SCs were treated with CT04 in the presence or absence of IGF-1 for 24 h. **(A,B)** Western blotting was performed to confirm the effect of IGF-1 on activation of AKT. GAPDH was used as the loading control (*n* = 4, *P < 0.05). **(C-L)** Analysis of EdU incorporation indicated that IGF-1 partly restored the declined EdU positive ratio in CT04 treated cells (*n* = 15, *P < 0.05). **(M)** Statistics of cell density displayed that CT04-mediated inhibition of SC density was reversed by IGF-1 (*n* = 15, *P < 0.05). **(N)** WST-1 assay revealed that IGF-1 treatment partly restored the change in absorbance value caused by CT04 (*n* = 4, *P < 0.05). **(O)** Western blots showed that application of IGF-1 up-regulated the expression of PCNA in the CT04 treated cells. The blots were cropped from different parts of the same gel. **(P)** Statistical diagram of relative protein expression of PCNA (*n* = 4, *P < 0.05). The expression levels of target proteins in the control group were normalized to 1.

**Figure 7:**
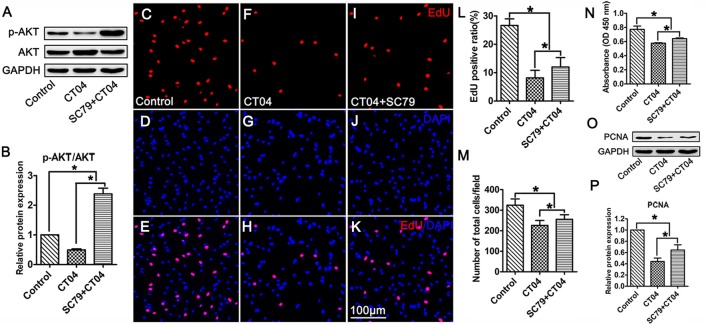
Activation of AKT by SC79 counteracts the inhibitory effect of CT04 on SC proliferation. SCs were treated with DMSO as vehicle control. The SCs were treated with CT04 with or without SC79 for 24 h. **(A,B)** Western blotting was performed to confirm the effect of SC79 on activation of AKT (*n* = 4, *P < 0.05). **(C-L)** The EdU incorporation assay suggested that application of SC79 increased the EdU positive ratio in CT04 treated cells (*n* = 15, *P < 0.05). **(M)** The statistics showed that CT04-mediated suppression of SC density was partly restored by SC79 (*n* = 15, *P < 0.05). **(N)** Assessment of cell proliferation by WST-1 assay displayed that SC79 counteracted the inhibitory effect of CT04 on SC proliferation (*n* = 4, *P < 0.05). **(O,P)** Western blots and statistical data indicated that the addition of SC79 increased the expression of PCNA in the CT04 treated cells (*n* = 4, *P < 0.05). The blots were cropped from different parts of the same gel. The expression levels of target proteins in the control group were normalized to 1.

To ascertain the potential role of AKT pathway on CT04-mediated inhibition of SC proliferation, we treated the SCs with CT04 in the presence or absence of AKT activators (IGF-1 (Jo et al., 2012; Sabater et al., 2017) or SC79 (Wu et al., 2017; Yang et al., 2018; Zhou et al., 2018)), respectively. Western blotting indicated that IGF-1 dramatically increased the level of p-AKT while the level of total AKT was not affected (Figures 6A,B). Subsequently, the EdU assay showed that CT04-induced suppression of EdU positive ratio of SCs and the cell density were obviously restored by IGF-1 (Figures 6C–L,M). The effect of IGF-1 on SC proliferation was also verified by the change in absorbance value detected by WST-1 measurement (Figure 6N). What’s more, the down-regulation of PCNA expression caused by CT04 treatment was also significantly alleviated by IGF-1 as shown in western blots (Figures 6O,P). Similar findings were observed when SCs were treated with CT04 in the presence of SC79, another specific activator of AKT. The results of cell density, EdU, WST-1 and western blotting assays also revealed that the addition of SC79 partly reversed CT04-mediated suppression of SC proliferation (Figure 7).

## Discussion

Collectively, overall data of the present study demonstrate that: (1) data got from the assessments of cell density, EdU, WST-1 and PCNA expression indicate that the inhibition of RhoA-subfamily GTPases by C3 transferase (CT04) can suppress the SC proliferation; (2) Live/Dead cell staining assay indicates CT04 does not induce cell death in the SCs cultures, which means the effect of CT04 on SC proliferation was not related to the cytotoxicity; (3) Y27632 (a widely used specific ROCK inhibitor) does not affect the SC proliferation. By which excludes the possibility of RhoA-subfamily GTPases regulating SC proliferation via ROCK pathway; (4) the level of p-AKT is significantly decreased in the CT04 treated SCs, while the total AKT is unaffected. Since AKT activation (phosphorylation) is involved in regulating the proliferation of many kinds of cells including SCs, these results suggest AKT inactivation might play a role in CT04 negative effects on SC proliferation; (5) CT04 treatment results in down-regulation of PI3K and up-regulation of PTEN, which can confirm and verify the results of CT04 suppressing the AKT activation; and (6) reversing the AKT activation by IGF-1 or SC79 (two kinds of widely used AKT activators) can significantly alleviate the inhibitory effect of CT04 on SC proliferation. These data confirm that AKT pathway is involved in the mechanisms of CT04-induced suppression on SC proliferation.

Since SCs are important glial cells in peripheral nerve and they have attractive application prospects in cell transplantation for the therapy of nervous system injury, the biology and application of SCs have been attracting a great deal of attention (Belin et al., 2017). Recent decades, lots of publications focused on the study of SC proliferation (Atanasoski et al., 2004; Deng et al., 2017; Pan et al., 2017; Piñero et al., 2017). As molecular switches that control the organization and dynamics of the actin cytoskeleton, Rho GTPases are considered to be key regulators of proliferation of various cells (Hu et al., 2014; Wang et al., 2014). Our previous study indicated that lentivirus-mediated RhoA knocking down to reduce the total protein level of RhoA could significantly slack the proliferation of SCs (Wen et al., 2017a). In this study, we aimed to reveal whether inhibiting the activation of RhoA-subfamily GTPases can impact the SC proliferation. CT04 is able to specifically and irreversibly inhibit activation of RhoA-subfamily GTPases by ADP ribosylation, our recent study proved CT04 treatment could reduce the level of activated GTP-RhoA in cultured SCs (Wen et al., 2018). Combined with the present data of cell density, EdU assay, WST-1 assessment and western blotting of PCNA, we can safely draw the conclusion that inhibition of RhoA-subfamily GTPases by CT04 can significantly slow down the SC proliferation.

Up to now, the most well-known downstream effector of RhoA-subfamily GTPases is ROCK. In existing reports, RhoA subfamily plays roles in various biologic effects including cell proliferation always by regulating actin dynamics via ROCK pathway. Thus, we naturally consider CT04 may also influence SC proliferation through ROCK pathway. However, the treatment of Y27632, a widely used inhibitor of ROCK, has no significant impact on the parameters of SC proliferation including cell density, EdU, WST-1 and western blotting of PCNA. Therefore, ROCK is impossible taking part in the regulation of RhoA subfamily on SC proliferation.

AKT pathway is demonstrated as an important signaling pathway in the regulation of cell proliferation, including SCs (Wu et al., 2016; Liu et al., 2017; Zhao et al., 2017). This drew us to think whether CT04 treatment modulated SC proliferation via AKT pathway. In order to test this hypothesis, the phosphorylation of AKT was firstly evaluated. The results demonstrated that CT04 treatment significantly reduced the level of p-AKT. Moreover, the expression level of PI3K, which is the crucial positive upstream molecule of AKT, was markedly decreased. It has been shown in several researches that in the PTEN/PI3K/AKT signaling pathway, PI3K can catalyze 3,4,5-phosphatidylinositol trisphosphate phosphorylation and then activate AKT to promote the proliferation of cells (Zhang et al., 2015). In addition, we found that the expression of PTEN was dramatically up-regulated. PTEN is an important negative regulator of AKT pathway, it can antagonize PI3K and then weaken the activation of AKT (Zhang et al., 2015; Ahmed et al., 2016). Therefore, the up-regulation of PTEN and down-regulation of PI3K further confirmed that AKT pathway could be inactivated by CT04. To further validate whether the inactivation of AKT pathway was responsible for CT04-mediated suppression of SC proliferation, two kinds of activators of AKT pathway (IGF-1 and SC79) were used to do the rescue experiments. As expected, both IGF-1 and SC79 could reverse the AKT inactivation by CT04 and significantly alleviate the inhibitory effect of CT04 on SC proliferation.

Taken together, we can conclude that: (1) C3 transferase (CT04) treatment can significantly suppress the SC proliferation. Considering SCs are important glial cells in peripheral nerves and C3 transferase is widely used to promote axonal regeneration in the injured peripheral nerve, the present study indicates further studies are needed to explore new strategies to avoid this side-effect when using this drug to treat the injured nerve; and (2) the effect of CT04 on SC proliferation involves the AKT pathway. While ROCK, the most well-known downstream molecule of Rho subfamily, is independent of the effect of CT04 on the SC proliferation. Considering the complicate signaling networks of Rho GTPases in most kinds of cells, and combining with present data, we believe that AKT is not the only downstream of RhoA subfamily on SC proliferation. The clear mechanism needs further studies in the future.

## Author Contributions

JG and DT designed the study. DT, JW, LL and XW performed experiments. CQ, MP and ML participated in collecting and analyzing experimental data. JD, XH and HZ contributed for double blind analyzing data and doing statistics. The manuscript was written by JG, DT and reviewed by all authors.

## Conflict of Interest Statement

The authors declare that the research was conducted in the absence of any commercial or financial relationships that could be construed as a potential conflict of interest.
